# Genetic and methylation variation in the CYP2B6 gene is related to circulating *p,p*′-dde levels in a population-based sample

**DOI:** 10.1016/j.envint.2016.11.010

**Published:** 2017-01

**Authors:** Lars Lind, Esther Ng, Erik Ingelsson, Cecilia Lindgren, Samira Salihovic, Bert van Bavel, Anubha Mahajan, Erik Lampa, Andrew P. Morris, P. Monica Lind

**Affiliations:** a Department of Medical Sciences, Cardiovascular Epidemiology, Uppsala University, Uppsala, Sweden; b Wellcome Trust Centre for Human Genetics, University of Oxford, Oxford, UK; c Department of Medical Sciences, Molecular Epidemiology and Science for Life Laboratory, Uppsala University, Uppsala, Sweden; d Department of Medicine, Division of Cardiovascular Medicine, Stanford University School of Medicine, Stanford, CA, USA; e Broad Institute of the Massachusetts Institute of Technology and Harvard University, Cambridge, USA; f MTM Research Centre, School of Science and Technology, Örebro University, Sweden; g Department of Biostatistics, University of Liverpool, Liverpool, UK; h Department of Medical Sciences, Occupational and Environmental Medicine, Uppsala University, Sweden

**Keywords:** GWAS, CYP2B6, DDE, Metabolism, Methylation

## Abstract

**Objectives:**

Since the metabolism of the organochlorine pesticide dichlorodiphenyltrichloroethane (DDT) is not fully known in humans, we evaluated if circulating levels of a major breakdown product of DDT, *p,p*′-DDE, were related to genome-wide genetic and methylation variation in a population-based sample.

**Methods:**

In the population-based Prospective Investigation of the Vasculature in Uppsala Seniors (PIVUS) study (1016 subjects all aged 70), circulating levels of *p,p*′-DDE were analyzed by high-resolution chromatography coupled to high-resolution mass spectrometry (HRGC/HRMS). Genetic variants were genotyped and imputed (1000 Genomes reference, March 2012 release). Methylation sites were assayed using the Illumina HumanMethylation450 array in whole blood. A genome-wide association study (GWAS) approach was applied.

**Results:**

Evidence for genome-wide significant association with *p,p*′-DDE levels was observed only for a locus at chromosome 19 corresponding to the *CYP2B6* gene (lead SNP rs7260538). Subjects being homozygote for the G allele showed a median level of 472 ng/g lipid, while the corresponding level for those being homozygote for the T allele was 192 ng/g lipid (*p* = 1.5 × 10^− 31^). An analysis conditioned on the lead SNP disclosed a distinct signal in the same gene (rs7255374, position chr19:41520351; *p* = 2.2 × 10^− 8^).

A whole-genome methylation analysis showed one significant relationship vs. *p,p*′-DDE levels (*p* = 6.2 × 10^− 9^) located 7 kb downstream the *CYP2B6* gene (cg27089200, position chr19:41531976). This CpG-site was also related to the lead SNP (*p* = 3.8 × 10^− 35^), but mediated only 4% of the effect of the lead SNP on *p,p*′-DDE levels.

**Conclusion:**

Circulating levels of *p,p*′-DDE were related to genetic variation in the *CYP2B6* gene in the general elderly population. DNA methylation in this gene is not closely linked to the *p,p*′-DDE levels.

## Highlights

• A whole genome scan was used to investigate the metabolic route of *p,p*′-DDE in human.
• Genetic variation in the CYP2B6 gene was associated with *p,p*′-DDE levels.
• Methylation close to the same gene was associated with *p,p*′-DDE levels.
• Modern genetic analyses showed CYP2B6 to be linked to *p,p*′-DDE levels in human.

## 1 Introduction

Dichlorodiphenyltrichloroethane (DDT) is an insecticide heavily used since the second half of World War II. Due to reproductive problems observed in wild animals, DDT was banned in the 1970s and 1980s in most high-income countries. Despite its toxic properties, DDT is still used in many developing countries, mainly to fight malaria (Eskenazi et al., 2009). A major metabolite of DDT is 2, 2-*bis* (4-chlorophenyl)-1, 1-dichloroethene (*p,p*′-DDE). *p,p*′-DDE is highly lipophilic and thereby accumulates in adipose tissue and has been estimated to have a half-life of 10–15 years. Although DDT is not used as a pesticide any longer in high-income countries, due to its persistence in the environment and accumulation in the food chain in fish and meat, there is still exposure taking place. However, this exposure is not as high as before the ban. An accumulation occurs with ageing, so higher levels of DDT/*p,p*′-DDE are seen in older subjects than in younger individuals as a result from a continuous cumulative exposure (Ye et al., 2015). We have recently reported that high levels of *p,p*′-DDE are related to prevalent obesity, diabetes and hypertension (Lee et al., 2011, Lind et al., 2014b, Ronn et al., 2011).

Several studies have shown that DDT alters the activity of many microsomal enzymes, including those involved in phase I and phase II metabolism of xenobiotics (Lubet et al., 1992, Madhukar and Matsumura, 1979) in different species (Abernathy et al., 1971, Bunyan et al., 1972, Henneman et al., 1994, Li et al., 1995, Lubet et al., 1990). Pharmacodynamic studies on CYP2B induction indicated no important differences between the isomer *p,p*′-DDT and its metabolite DDE (Nims et al., 1998). The effects consisted mainly of an induction of the CYP2B subfamily, a lesser induction on CYP3A, and minimal or no induction of CYP1A. On this basis, DDT has been considered a phenobarbiturate-type of inducer (Nims et al., 1998, Okey, 1972).

Toxicokinetic studies of persistent organic pollutants, such as DDT/DDE, have mainly been conducted in an experimental setting (as cited above), and therefore such results have to be validated in humans. One way to perform such studies in humans is to relate functional genetic variations in genes known to be involved in the kinetics of environmental contaminants to levels of the contaminant of interest, like relating levels of polychlorinated biphenyls to single nuclear polymorphisms (SNP) in the CYP1A1 gene (Lind et al., 2014a). However, such an approach demands a detailed prior knowledge of the metabolism of the compound of interest. Another approach is to use a genome-wide association study (GWAS), which test a great number of SNP across the genome without a prior hypothesis. We have previously used the GWAS approach and found that circulating levels of several of the PCBs were related to variation in the CYP2B6 gene (Ng et al., 2015b), and whole blood manganese levels to be related to variation in the SLC39A8 and SLC30A10 genes, while mercury and cadmium levels shared associations with variation in other genes (Ng et al., 2015a).

The expression of a protein is not only governed by the variation in base-pairs in the genes, but also by epigenetic mechanisms, like methylation. For example, it has been shown that *p,p*′-DDE levels are linked to alterations in global DNA methylation (Rusiecki et al., 2008).

Since the metabolism of DDT and *p,p*′-DDE has mainly been studied in the experimental setting, we used the GWAS approach to relate genetic variation to circulating levels of *p,p*′-DDE. For this purpose, we used data from the Prospective Investigation of the Vasculature in Uppsala Seniors (PIVUS) study (Lind et al., 2005), in which extensive genotyping has been performed, together with measurements of circulating *p,p*′-DDE levels. We also investigated if *p,p*′-DDE levels were related to differential methylation using a whole-genome approach.

## 2 Material and methods

### 2.1 Subjects

The PIVUS study was originally designed to study markers of subclinical cardiovascular disease as risk factors for incident cardiovascular diseases (Lind et al., 2005). Eligible subjects were all aged 70 and lived in the community of Uppsala, Sweden, with a total population of approximately 175,000 individuals. The subjects were randomly chosen from the register of community living kept by the City council of Uppsala. Of a total of 2027 invited individuals (50% being females), 1016 subjects participated, giving a participation rate of 50.1%. The study was approved by the Ethics Committee of the University of Uppsala and all the participants gave their informed consent prior to the study.

All subjects were investigated in the morning after an overnight fast. No medication or smoking was allowed after midnight. The participants were asked to answer a questionnaire about their medical history, smoking habits and regular medication. Blood samples for determinations of *p,p*′-DDE levels, genotyping and DNA methylation were drawn at the same time in the fasting state at the age of 70 years. *p,p*′-DDE levels were measured in serum and DNA was prepared from leukocytes in whole blood specimens.

### 2.1 *p,p*′-DDE analyses

*p,p*′-DDE levels were measured in stored serum samples using a Micromass Autospec Ultima (Waters, Milford, MA, USA) high-resolution gas chromatography coupled to a high resolution mass spectrometry (HRGC/HRMS) system based on the method by Sandau and colleagues (Barr et al., 2003) with some modifications. A more detailed description of the analysis in this sample has previously been presented (Salihovic et al., 2012).

### 2.2 Genotyping and imputation

Genotyping was performed on all participants using the Illumina Metabochip together with the Illumina OmniExpress chip. Samples were excluded based on call rate < 95%, extreme heterozygosity (> 3 SD from the mean), gender discordance, duplicated samples, close relatives or ethnic outliers. Variants with exact Hardy-Weinberg equilibrium (HWE) *p*-value < 1 × 10^− 6^, call rate < 0.99, SNPs with minor allele frequency [MAF] < 5% were excluded from the scaffold prior to imputation. The cleaned genotype data were imputed up to the 1000 Genomes, March 2012 release reference panel (multi-ethnic panel on NCBI build 37 [b37]) using IMPUTE v.2.2.2.

### 2.3 Regional DNA methylation

Methylation sites across the genome were assayed using the Illumina HumanMethylation450k Beadchip, which detects methylation based on genotyping of bisulfite-converted genomic DNA, covering 482,421 CpG-sites and 3091 non-CpG sites. Samples were excluded based on call rate (98.5% probes with detection *p*-value < 0.01), leukocyte count > 10 (× 10^9^ cells/L), bisulfite conversion efficiency outliers, or more than one mismatch when comparing the SNPs on the methylation chip and the Omni/Metabochip genotyping chips. Data on the X and Y chromosomes were not used in the analysis. A quantile normalization of the signal intensities was performed per individual and undertaken separately for type-I and type-II probes of the chip. Beta-values were then calculated as the percentage methylation at a site, denoted degree of methylation in the text. A total of 20,522 methylation sites were excluded from the analysis since their probes mapped to multiple locations in the genome with at least two mismatches, in accordance with methods used by other investigators (Grundberg et al., 2013).

### 2.4 Statistical analyses

Since not normally distributed, *p,p*′-DDE was natural log-transformed before analysis, and used in the further analysis as a continuous variable. Since no priori hypotheses regarding which genes that could be related to *p,p*′-DDE levels were given in the present study, a GWAS with *p,p*′-DDE as dependent variable was performed using the score-based test in SNPTEST 2.4.1 (Marchini et al., 2007), accounting for imputation uncertainty in a missing data likelihood and assuming an additive genetic effect. Only SNPs with MAF > 0.05 and IMPUTE2 info > 0.4 were included in the analysis. Gender and two principal components (based on the genetic structure in the sample, here used to adjust for any genetic heterogeneity within the sample) were included as covariates and a *p*-value < × 10^− 8^ defined a genome-wide significant finding.

The genomic inflation factor lambda (Devlin and Roeder, 1999) was calculated as a quality control to assess the evidence for residual population structure that was not accounted for in the association analysis. Even studies with relatively homogeneous populations are susceptible to residual confounding by population stratification.

The associations between SNPs within and around *CYP2B6* (10 kb up and downstream of the transcript boundaries on b37 obtained by the UCSC Table Browser [http://genome.ucsc.edu/cgi-bin/hgTables]) were visualized using the program LocusZoom 1.1 (Pruim et al., 2010). Following identification of the most significantly associated SNP in the analysis, a conditional analysis was performed (including genotypes at this lead SNP with an additive genetic effect in the regression model) to identify additional distinct signals within or around *CYP2B6*. The “beta” referred to in the following text and tables is the regression coefficient in linear regression analysis. Thus, a beta for a SNP as an independent variable is the contribution of an additional allele.

Linear regression was used to analyze the relationships between the degree of methylation at sites across the genome and *p,p*′-DDE levels, adjusting for gender, leukocyte cell fractions according to Houseman (Houseman et al., 2012) sample batch, bisulfite conversion efficiency, storage time and smoking status (never, previous, current). Probes with a SNP within 10 bp were filtered out as were probes with known genomic variants with a minor allele frequency of < 1% according to the HumanMethylation450 annotation files. A Bonferroni-adjustment was used accounting for 322,756 tested methylation sites (critical *p*-value 1.55 × 10^− 7^). An interaction term (multiplicative) between the lead methylation site and sex regarding *p,p*′-DDE levels were used to evaluate any sex-differences in the lead methylation site vs. *p,p*′-DDE levels relationship.

The GWAS and whole-genome methylation analysis were performed as two separate analyses.

The relations between the 2046 SNPs within ± 0.5 Mb (predictors) and the lead methylation site were also analyzed by linear regression analysis (*p*-value 2 × 10^− 5^), including confounders stated above. In studies using both SNPs and methylation it is possible test the causal pathway: SNP- > methylation- > outcome to see if the association between a SNP and an outcome (like *p,p*′-DDE) is mediated by the methylation. Structural equation modeling (SEM) (Westland, 2015) was used to evaluate the degree of mediation of the lead methylation site on the relationship between the lead SNP and *p,p*′-DDE levels. Model used: (lead SNP- > *p,p*′-DDE levels) (lead SNP- > lead methylation site) (lead methylation site- > *p,p*′-DDE levels), where arrows indicate linear regression.

## 3 Results

Basic characteristics regarding diseases and medications are given in Table 1.

### 3.1 *p,p*′-DDE levels and SNPs in the CYP2B6 gene

**Fig. 1:**
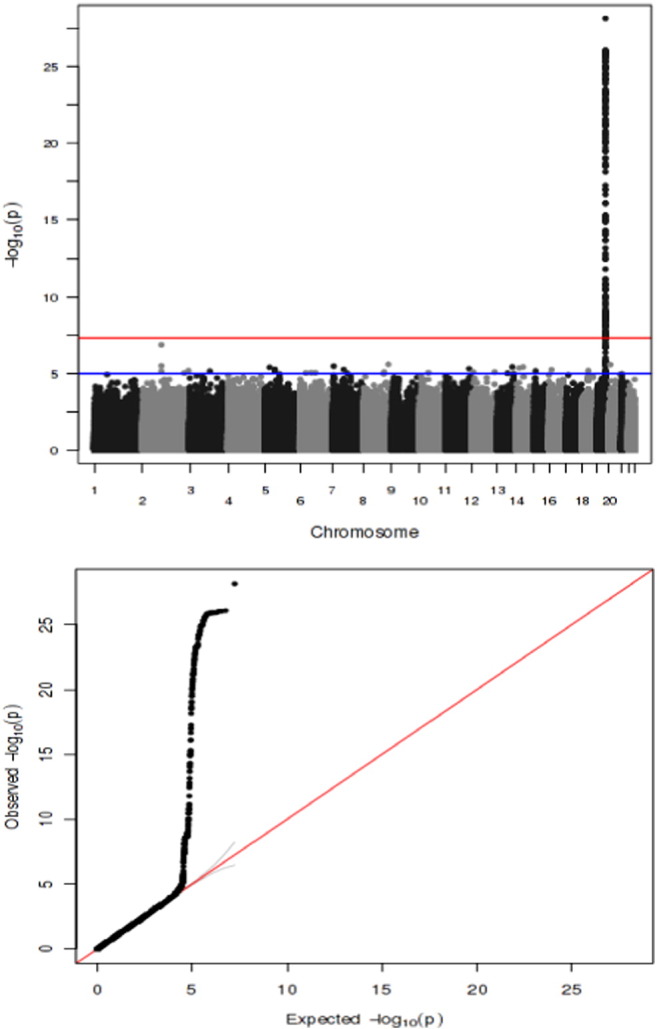
Manhattan plot for a genome wide association study (GWAS) for plasma *p,p*′-DDE levels (upper panel) with − 10log *p*-value at the y-axis and the gross position in the genome at the x-axis. A Manhattan plot shows a point for each single nucleotide polymorphism (SNP) that has been tested in a unique linear regression model vs. *p,p*′-DDE levels. The red line indicates the GWAS level of significance (*p* < 5 ∗ 10^− 8^).

In the GWAS, we did not observe any evidence of systematic inflation of *p*-values reflecting residual population structure (Fig. 1, lower panel; lambda, 1.01). Evidence for genome-wide significant association with *p,p*′-DDE levels was observed only for one locus at chromosome 19 corresponding to the *CYP2B6* gene (Fig. 1, upper panel).

**Fig. 2:**
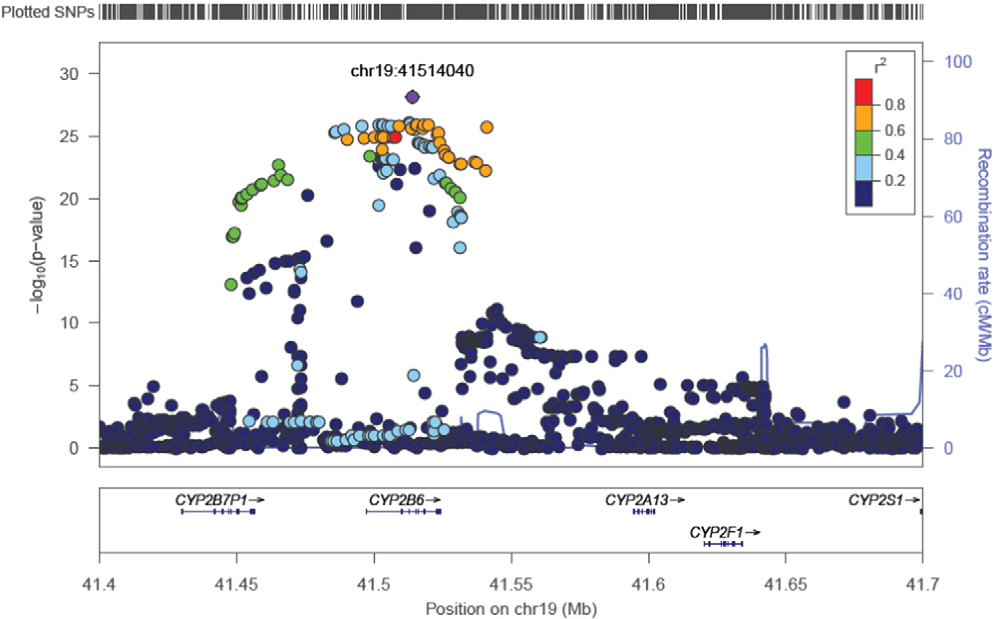
Regional plot for linear regression analysis regarding associations between *p,p*′-DDE levels and single nucleotide polymorphisms (SNPs) in or close to the *CYP2B6* gene. The -10log *p*-values for the associations are given at the y-axis and the positions at chromosome 19 are given at the x-axis. This figure is in fact only a zoom-in of the region of interest on chromosome 19 that is given in the Manhattan plot in Fig. 1. The lead SNP is indicated by the grey diamond and the color of the other SNPs denotes the linkage disequilibrium (r^2^) (meaning the relationship between the different SNPs in the region) in relation to the top hit.

**Fig. 3:**
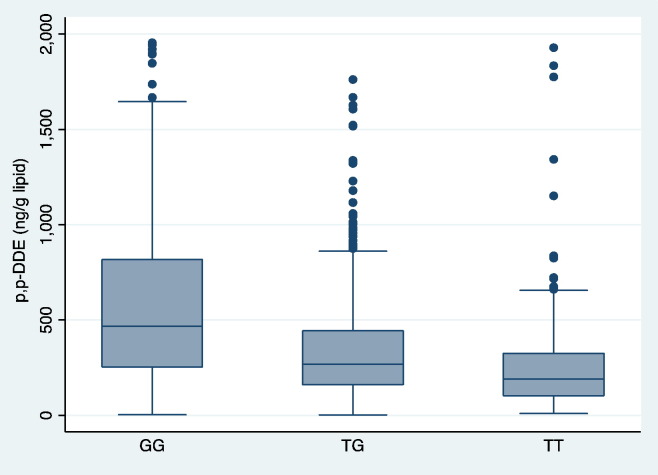
Box plot showing *p,p*′-DDE levels for the different genotypes of rs7260538 at chromosome 19. Values > 2000 are not shown.

A regional plot of the 769 SNPs within or close to *CYP2B6* gene (chr 19, position range 41.4–41.6 Mb) is presented in Fig. 2. The lead SNP, rs7260538 at position chr19:41514040, 19q13.2, is located in the intron region of NM_000767.4 (minor allele frequency 0.44, Hardy-Weinberg equilibrium 0.89, *p* = 1.5 × 10^− 31^). As could be seen in Fig. 3, subjects being homozygote for the G allele showed a median level of 472 ng/g lipid, while the corresponding level for those being homozygote for the T allele was 192 ng/g lipid (*p* = 1.5 × 10^− 31^). Adjusting for BMI did only alter this relationship to a very minor degree and the data given above is without BMI-adjustment.

An analysis conditioned on the lead SNP disclosed a distinct signal (rs7255374, position chr19:41,520,351) being associated with *p,p*′-DDE (*p* = 2.2 × 10^− 8^; Supplementary Fig. 1, upper panel). This secondary signal is also located in the intron region of NM_000767.4. No further significant signals were found in an analysis conditioned on both of these signals (see Supplementary Fig. 1, lower panel). If both the lead SNP and this secondary signal entered a regression model as independent variables and *p,p*′-DDE as dependent variable, these two SNPs explained 17% of the variation in *p,p*′-DDE levels in PIVUS, as determined from the R^2^-value given in the regression model (data not shown in tables).

In Suppl. Table 1, all associations with *p* < 5 ∗ 10^− 8^) are shown.

*CYP2B6* has 28 known alleles (http://www.cypalleles.ki.se/cyp2b6.htm). The most common of the haplotypes is CYP2B6*6 (frequency 0.038 in the present sample). This haplotype, defined by two SNPs (rs3745274, Chr19:41512841 and rs2279343, chr19:41515263), was related to *p,p*′-DDE levels in our sample (*p* = 0.00046). However, in an analysis conditioned on the two SNPs defining the CYP2B6*6 haplotype, several of the other SNPs in the *CYP2B6* region were still significantly related to *p,p*′-DDE levels (*p* < 10^− 10^ for the most strongly associated variant, Supplementary Fig. 2).

### 3.2 *p,p*′-DDE levels and methylation in the CYP2B6 gene

**Fig. 4:**
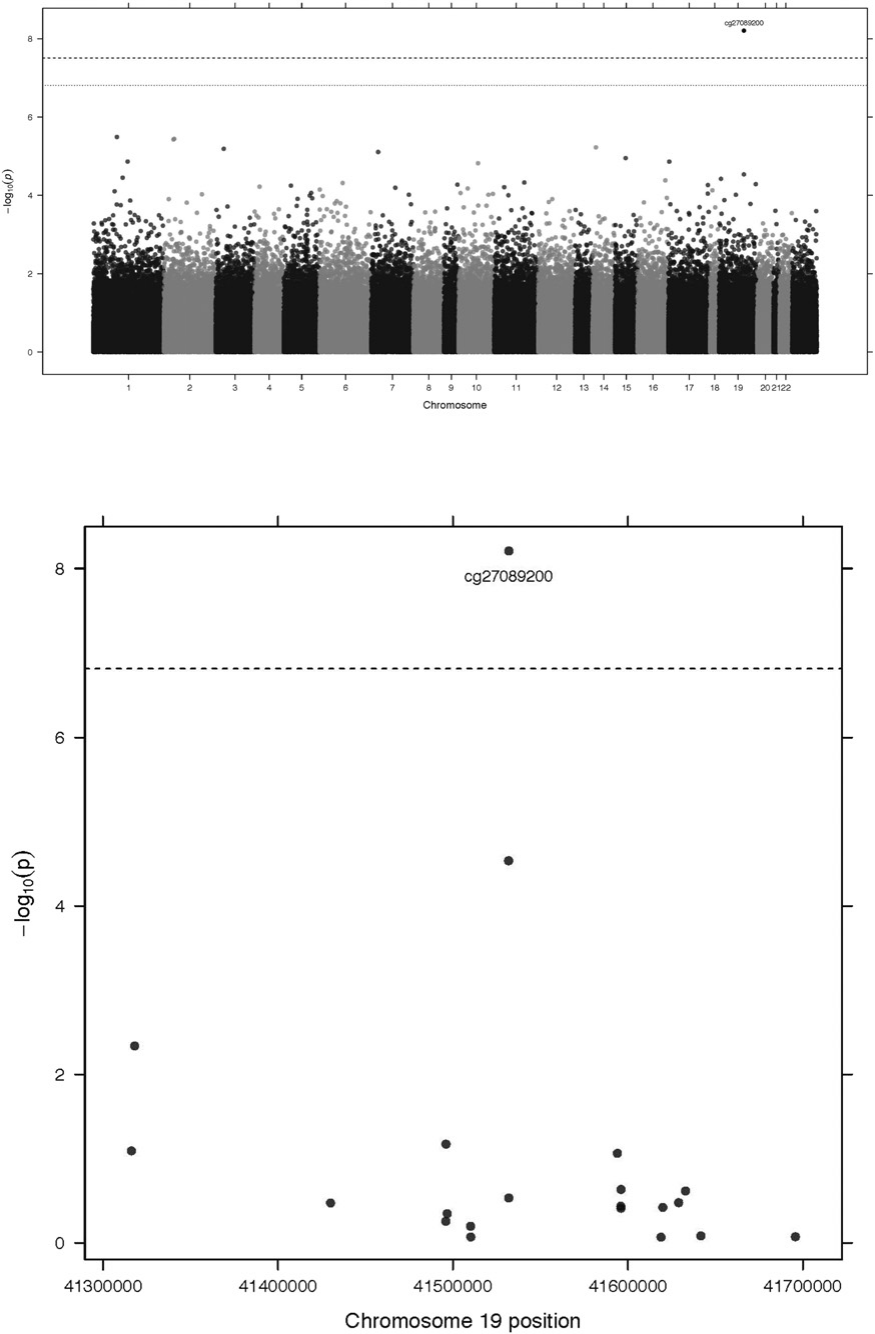
Manhattan plot for a whole-genome methylation analysis of plasma *p,p*′-DDE levels in relation to ~ 450 k methylation sites (upper panel) with − 10log *p*-value at the y-axis and the gross position in the genome at the x-axis. The lower panel shows a regional plot for linear regression analysis regarding associations between *p,p*′-DDE levels and methylation loci in or close to the *CYP2B6* gene. The -10log *p*-values for the associations are given at the y-axis and the positions at chromosome 19 are given at the x-axis. The lead methylation site in this analysis showed a *p*-value of 6.2 ∗ 10^− 9^.

A whole-genome methylation analysis suggested an association between one loci at chromosome 19 and *p,p*′-DDE levels (beta 0.12, SE 0.021, *p* = 6.2 ∗ 10^− 9^, fig. 4). According to that regression model, a change in 400 ng/g lipids in *p,p*′-DDE (the interquartile range) corresponds to a change in methylation at that loci by 0.0091. Adding BMI as a confounder to the model did only change the result marginally.

In Suppl. Table 2, all association with a *p*-value < 10^− 5^ are shown, including regression coefficients (beta) and SE.

This lead methylation site being located approximately 7 kb downstream the CYP2B6 gene (cg27089200, position chr19:41531976, mean value 0.25 (SD 0.07)) was also related several SNPs in this region (most significant SNP in this analysis was rs7255904, beta 0.50, SE 0.020, *p* = 1.3 × 10^− 101^). This methylation site was associated with the lead SNP (rs7260538) (beta − 0.32, SE 0.025, *p* = 3.8 × 10^− 35^, Suppl. Fig. 3).

When the relationship between the lead methylation site and *p,p*′-DDE levels was conditioned on the lead SNP, the lead methylation site was no longer significant (*p* = 0.25). A mediation analysis (SEM) showed that the lead methylation site only mediated 4% of the effect of the lead SNP on *p,p*′-DDE levels.

We investigated if any of the drug classes given in Table 1 could confound the relationships between *p,p*′-DDE levels and the lead SNP or the lead methylation site by including the 14 kinds of drugs in one regression model with the lead SNP and one model with the lead methylation site. However, adjustment for the 14 drugs did not substantially alter the relationships between SNP/methylation sites and *p,p*′-DDE levels described above.

When including an interaction term between *p,p*′-DDE levels (continuous) and sex in the regression model relating *p,p*′-DDE levels vs. the lead methylation site, no significant interaction was seen (*p* = 0.55). Therefore the regression coefficient (beta) was similar in men and women (0.15 in men and 0.11 in women).

The lower panel shows the corresponding Q-Q plot with the observed *p*-values (on a − 10log scale) from all the linear regression models using all the available SNPs plotted vs. the expected *p*-values (given from a simulation of > 1 million regression models). The red line denotes the line of identity.

## 4 Discussion

The present study showed that circulating levels of *p,p*′-DDE, a more stable metabolite of DDT, were related to genetic variation in the CYP2B6 gene. This finding is in accordance with experimental studies showing that DDT and its metabolite *p,p*′-DDE cause induction of CYP2B enzymes (Nims et al., 1998, Okey, 1972), but this is the first study to show this link between *p,p*′-DDE and CYP2B6 in humans. We found DNA methylation in this gene to not play a major role for the *p,p*′-DDE levels, since the impact of DNA methylation was minor when taken the lead SNP into account in the SEM analyses.

*p,p*′-DDE is still measurable in the circulation in almost all individuals in high-income countries (Salihovic et al., 2012). The median concentration of *p,p*′-DDE in this study of elderly individuals is similar to those previously reported from Sweden and Norway (Hagmar et al., 2006, Hardell et al., 2010, Sandanger et al., 2006). However, the levels of *p,p*′-DDE in the present study were about three times lower when comparing with elderly individuals from Belgium (Koppen et al., 2002) and the United States (NHANES 2003–2004) in samples collected during the same time period.

The CYP2B6 expression and activity is highly variable between individuals, owing to genetic polymorphisms. Regarding CYP2B6, interindividual variability exist at both mRNA and protein levels, and these differences are roughly over 100-fold (Wang and Tompkins, 2008). Several functionally relevant single-nucleotide polymorphisms (SNPs) were reported to affect CYP2B6 activity. Their most common functionally deficient allele is CYP2B6*6. The CYP2B6*6 homozygous carriers CYP2B6*6/*6, and its compound heterozygous genotypes CYP2B6*1/*6 were found to show poor metabolizer phenotypes for several drugs in vivo (Lang et al., 2001). It can therefore be assumed that SNPs in this gene could identify those with a poor metabolizing capacity of *p,p*′-DDE as well.

From the conditional analysis it is evident that there are at least two distinct association signals in the *CYP2B6* region related to *p,p*′-DDE levels. Since both of these SNPs are located in an intronic region, it is far from clear how these SNP might influence the *p,p*′-DDE levels, but together they explained 17% of the variation in *p,p*′-DDE levels in PIVUS, showing that CYP2B6 activity is of importance for the circulating levels of this organochlorine pesticide.

In contrast to the top hits in the *CYP2B6* region described here, it is known that the common CYP2B6*6 haplotype, encoding a change from glutamine to histidine in one amino acid position of the protein, could govern CYP2B6 activity in vitro. Also, the CYP2B6*6 haplotype was related to the *p,p*′-DDE levels, but when a further analysis was conducted conditioned on the SNPs in the CYP2B6*6 haplotype, a great number of other SNPs in the region were also significantly related to *p,p*′-DDE levels, suggesting that there is additional genetic variation of importance for *p,p*′-DDE levels in this region, beyond the known CYP2B6*6 haplotype. It should be noted that the frequency of the CYP2B6*6 haplotype was low, so the power to detect an independent contribution of this haplotype is limited.

The CYP2B6 enzyme is mainly expressed in the liver and in the brain in humans (Lamba et al., 2003) The expression of this enzyme in the liver is very important, since it has been estimated that CYP2B6 metabolizes about 25% of all pharmaceutical drugs on the market, and the expression is subject to a high degree of variability due to variation in the gene (Yang et al., 2013) Since DDT and *p,p*′-DDE are highly lipophilic they do accumulate in liver tissue and previously experimental studies and the present study show that variation in CYP2B6 is of major importance for the clearance of DDT/*p,p*′-DDE from the body in humans.

In addition to analyzing the relationships between *p,p*′-DDE levels and genetic variation in the *CYP2B6* gene, we also investigated how *p,p*′-DDE levels were associated with the degree of methylation in CpG-sites across the genome, since methylation could affect the activity of the enzyme. We found that the degree of methylation in one locus, 7 kb downstream of the *CYP2B6* gene was related to *p,p*′-DDE levels. This lead CpG-site was related to the lead SNP (and to other SNPs in that region), but since a further analysis showed that only 4% of the total effect of the lead SNP on *p,p*′-DDE levels was mediated by the lead methylation site, this effect seems to be of minor importance.

If an association between *p,p*′-DDE levels and a methylation site within or close to a gene is seen, as in the present study, two possible explanations exit. First, an SNP within or close to the gene governs the degree of methylation at that particular site and this SNP is also related to *p,p*′-DDE levels. In that case, the degree of methylation is a mediator of the effect of the SNP on *p,p*′-DDE levels. This can be tested by SEM analysis as performed in the present study.

Second, the methylation site is not related to the SNPs in that region, and therefore the effect of the methylation site on *p,p*′-DDE levels is independent of SNPs. In the present case, the lead methylation site was related to several SNPs in the region, including the lead SNP, and therefore the performed SEM analysis could conclude that the degree of methylation was of minor importance as compared to the effect of the SNP.

In the present study, it was disclosed that the association between the *p,p*′-DDE levels and the lead methylation site was in fact governed by a SNP. This finding is intriguing but it needs replication and corroboration by other studies in which genetic and epigenetic data are available.

Just as in our previous study on genetic variation and PCB levels (Ng et al., 2015b), we now found that the lead SNP is intronic. How intronic variation influence protein levels and function is not fully understood but several mechanisms are likely to exist. First, an intronic variant might be in linkage with a function SNP not measured or imputed. Second, genetic and epigenetic variants in introns can influence expression of different gene isoforms or alternative splicing. Third, alternative promoters for transcription of gene variants could be influenced by intronic variation.

The major strength of the present study is the large sample with both *p,p*′-DDE measurements, and extensive genotyping and methylation data. The limitations include that our study is conducted in elderly Caucasians, which limits the generalizability to other age- and ethnic groups, and the fact that we cannot replicate our findings in an independent sample, since we are not aware of other samples with both *p,p*′-DDE measurements and genetic information.

A power analysis taken place before this study was conducted showed that with approximately 1000 individuals in the study, we have a limited power to detect relationships where an exposure will explain < 4% of the outcome. Thus, it is not excluded that less powerful associations between *p,p*′-DDE and variation in other genes than CYP2B6 do exist.

The PIVUS study had a moderate participation rate of 50%. We have made an analysis of the non-participants and found a slightly higher prevalence of disabling disorders, such as stroke, but the prevalence's of many other diseases, like hypertension and myocardial infarction were similar in participants and non-participants (for details, see Lind et al., 2005). It is a well-known experience in population-based epidemiology that non-participants are somewhat more sick the participants. Since the aim of the present study was to investigate links between *p,p*′-DDE levels and genetics it will not be a disadvantage with a healthier sample than the underlying population, since diseases tend to obscure basic mechanisms of toxicokinetics.

It should also be acknowledged that a cross-sectional study has limitations regarding mediation analysis. Here we postulated that genetic variation- > methylation changes- > altered *p,p*′-DDE levels. Given the cross sectional nature other alternative pathways might exists, like *p,p*′-DDE- > altered methylation. Only longitudinal studies could solve those issues. Another limitation is that the results from the whole genome methylation assay were not validated by another type of assay.

In conclusion, genetic variation in the *CYP2B6* gene was related to circulating *p,p*′-DDE levels in the general elderly population. DNA methylation in this gene does not seem to play a major importance for the *p,p*′-DDE levels, but the fact that both an untargeted genome-wide genomic study and a methylation study linked *p,p*′-DDE levels to the CYP2B6 make it likely that CYP2B6 plays a major role in the metabolism of DDT in humans.

## Figures and Tables

**Table 1:** Self-reported history of cardiovascular (CV) disorders and regular drug intake given in percentage in the investigated sample (*n* = 1016). CABG/PCI = coronary revascularisation. N and percentage (in parenthesis) are given for each variable. For *p,p*′-DDE, median and 25th and 75th percentiles are given (in parenthesis).

Women	510 (50.2)
Myocardial infarction	72 (7.1)
Stroke	38 (3.7)
Angina pectoris	82 (8.1)
CABG/PCI	54 (5.3)
Congestive heart failure	39 (3.8)

Diabetes	88 (8.7)
Any regular drug	711 (70)
Any CV drug	457 (45)
Antihypertensive medication	325 (32)
Statins	152 (15)
Antidiabetic drugs	74 (7.3)
Aspirin/clopidogrel	182 (18)

Smoking	113 (11)
Obesity (BMI ≥ 30 kg/m^2^)	224 (22)
Education level: < 9 years 10–12 years > 12 years	579 (57) 182 (18) 255 (25)
*p,p*′-DDE (ng/g lipid)	308 (170–570)

## Appendix A Supplementary data

Supplementary material Image 1
