# Supplementary material for: Genetic and methylation variation in the CYP2B6 gene is related to circulating p,p′-dde levels in a population-based sample
Source: Environ Int. 2017 Jan;98:212–8. doi: 10.1016/j.envint.2016.11.010 (PMC5152752; doi:10.1016/j.envint.2016.11.010)
Supplement: Supplementary file 1 — Supplementary material [file mmc1.docx]

**Supplementary material for**

**GENETIC AND METHYLATION VARIATION IN THE CYP2B6 GENE IS RELATED TO CIRCULATING *p,p’-*DDE LEVELS IN A POPULATION-BASED SAMPLE**

Lars Lind^1*^, Esther Ng^2*^, Erik Ingelsson^3,4^, Cecilia Lindgren^2,5^, Samira Salihovic^3,6^, Bert van Bavel^6^, Anubha Mahajan^2^, Erik Lampa^1^, Andrew P. Morris^2,7^, P. Monica Lind^8^

**Supplementary Figure 1.** Regional association plot for linear regression analysis of associations between *p,p’*-DDE levels and SNPs in or close to the *CYP2B6* gene. The upper panel shows results from an analysis conditioned on the lead SNP (Chr19:41514040), while the lower panel shows an analysis conditioned on both the top hit and the secondary signal shown in the upper panel. The -10log p-values for the associations are given at the y-axis and the positions at chromosome 19 are given at the x-axis. The top hit is indicated by the grey diamond and the color of the other SNPs denotes linkage disequilibrium (r^2^) in relation to the top hit.

**Supplementary Figure 2.** Regional plot for linear regression analysis of associations between *p,p’*-DDE levels and SNPs in or close to the *CYP2B6* gene, following a conditional analysis of the two SNPs tagging the CYP2B6*6 haplotype (Chr19:41512841 and Chr19:41515263). The -10log p-values for the associations are given at the y-axis and the positions at chromosome 19 are given at the x-axis. The top hit is indicated by the grey diamond and the color of the other SNPs denotes linkage disequilibrium (r^2^) in relation to the top hit.

**Supplementary Figure 3.** Regional plot for linear regression analysis of associations between the degree of methylation at the lead methylation site (cg27089200) and SNPs in or close to the *CYP2B6* gene. The -10log p-values for the associations are given at the y-axis and the positions at chromosome 19 are given at the x-axis. The top hit is indicated by the grey diamond and the color of the other SNPs denotes linkage disequilibrium (r^2^) in relation to the top hit.


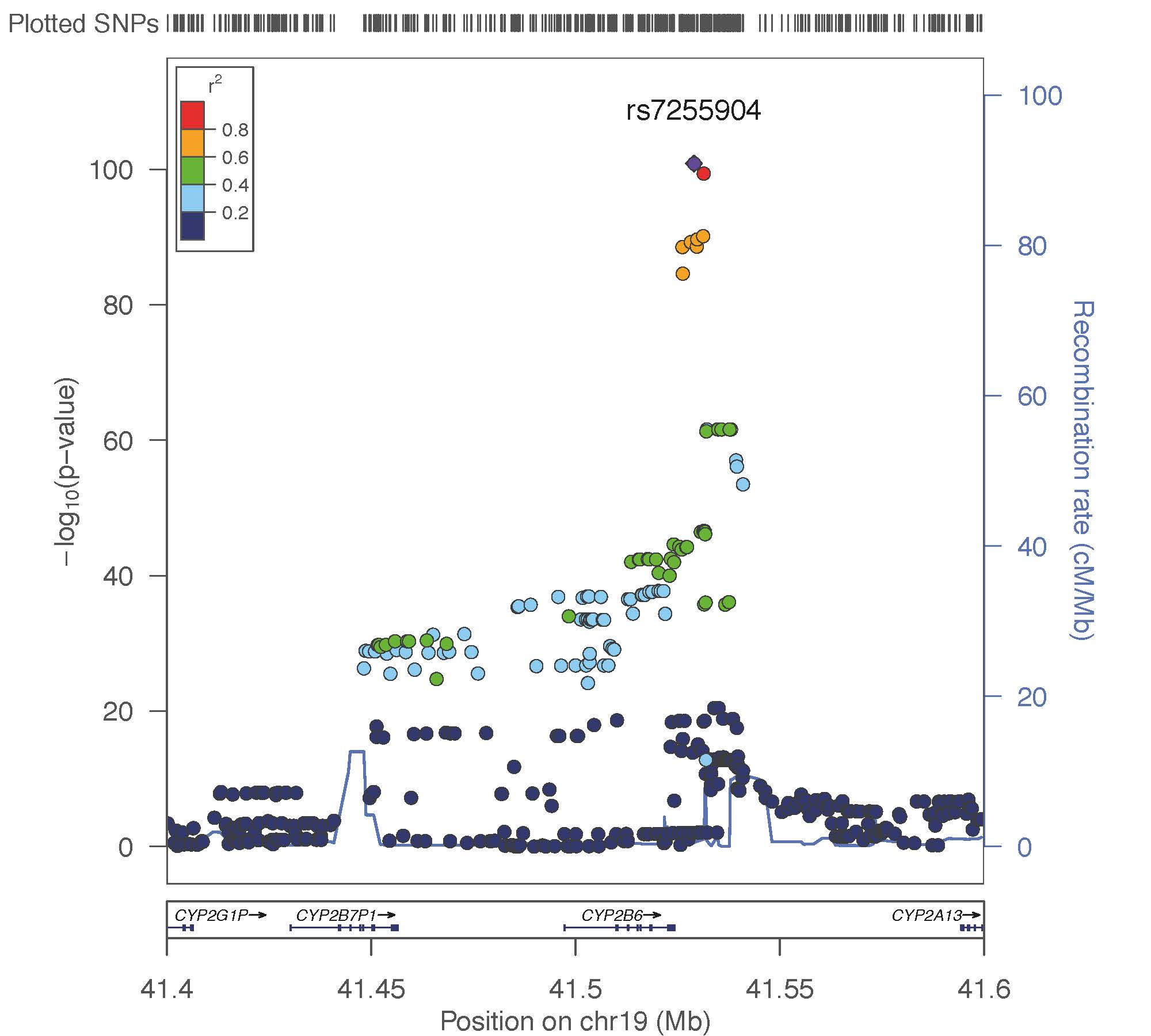


**Supplementary** **table 1.** Results from a genome-wide association analysis (GWAS) for serum levels of *p,p’-*DDE. Only SNPs with p-value < 10^-7^ are shown. All listed SNPs are in or close to the CYP2B6 gene. The Beta values are based on ln-transformed values of *p,p’-*DDE levels being used in the models. Since the minor allele is set to the lowest value in the linear regression model for the that particular SNP, a negative beta means highest levels of *p,p’-*DDE for the minor allele. e=*10^-^

| Chromosome | Position | SNP | Beta | SE | p-value |
| --- | --- | --- | --- | --- | --- |
| 19 | 41514040 | rs7260538 | -0.52 | 0.04 | 1.566e-31 |
| 19 | 41509033 | rs1987236 | -0.50 | 0.04 | 1.774e-28 |
| 19 | 41501716 | rs1872125 | 0.60 | 0.05 | 4.699e-28 |
| 19 | 41513385 | rs78313984 | 0.57 | 0.05 | 5.277e-28 |
| 19 | 41513445 | rs75446511 | 0.57 | 0.05 | 5.277e-28 |
| 19 | 41495755 | rs2054675 | 0.57 | 0.05 | 5.735e-28 |
| 19 | 41502834 | rs8104022 | 0.57 | 0.05 | 5.735e-28 |
| 19 | 41503257 | rs62109048 | 0.57 | 0.05 | 5.735e-28 |
| 19 | 41506191 | rs3786547 | 0.57 | 0.05 | 6.699e-28 |
| 19 | 41512841 | rs3745274 | 0.57 | 0.05 | 7.797e-28 |
| 19 | 41490404 | rs10418990 | -0.49 | 0.04 | 1.262e-27 |
| 19 | 41513613 | rs7257042 | -0.49 | 0.04 | 1.623e-27 |
| 19 | 41496461 | rs4802101 | -0.49 | 0.04 | 1.879e-27 |
| 19 | 41499989 | rs2014141 | -0.49 | 0.04 | 1.879e-27 |
| 19 | 41502602 | rs6508964 | -0.49 | 0.04 | 1.879e-27 |
| 19 | 41506953 | rs10412702 | -0.49 | 0.04 | 1.879e-27 |
| 19 | 41508020 | rs4803417 | -0.49 | 0.04 | 1.879e-27 |
| 19 | 41515483 | rs2279344 | -0.49 | 0.04 | 2.853e-27 |
| 19 | 41515702 | rs2279345 | -0.49 | 0.04 | 2.853e-27 |
| 19 | 41518007 | rs6508966 | -0.49 | 0.04 | 2.853e-27 |
| 19 | 41519715 | rs11671243 | -0.49 | 0.04 | 2.853e-27 |
| 19 | 41517688 | rs6508965 | -0.48 | 0.04 | 5.352e-27 |
| 19 | 41485821 | rs8106966 | 0.56 | 0.05 | 8.972e-27 |
| 19 | 41486066 | rs8106911 | 0.56 | 0.05 | 8.972e-27 |
| 19 | 41489004 | rs11083595 | 0.56 | 0.05 | 8.972e-27 |
| 19 | 41516182 | rs2306606 | 0.55 | 0.05 | 1.394e-26 |
| 19 | 41516349 | rs34906003 | 0.55 | 0.05 | 1.394e-26 |
| 19 | 41520297 | rs7255561 | 0.54 | 0.05 | 2.211e-26 |
| 19 | 41516946 | rs34830389 | 0.54 | 0.05 | 2.405e-26 |
| 19 | 41516950 | chr19:41516950:D | 0.54 | 0.05 | 2.405e-26 |
| 19 | 41518078 | rs7246456 | 0.54 | 0.05 | 2.405e-26 |
| 19 | 41518773 | rs8192719 | 0.54 | 0.05 | 2.405e-26 |
| 19 | 41520844 | rs11673270 | 0.54 | 0.05 | 2.474e-26 |
| 19 | 41521532 | rs7259965 | 0.54 | 0.05 | 2.474e-26 |
| 19 | 41523303 | rs7246465 | -0.48 | 0.04 | 4.164e-26 |
| 19 | 41525952 | rs1552223 | -0.46 | 0.04 | 1.316e-24 |
| 19 | 41531487 | rs11670798 | -0.47 | 0.04 | 1.336e-24 |
| 19 | 41531825 | rs3745276 | -0.47 | 0.04 | 1.521e-24 |
| 19 | 41501303 | rs59243457 | 0.53 | 0.05 | 1.662e-24 |
| 19 | 41502522 | rs8101756 | 0.53 | 0.05 | 1.662e-24 |
| 19 | 41502970 | rs10409285 | 0.53 | 0.05 | 1.662e-24 |
| 19 | 41503622 | rs7250873 | 0.53 | 0.05 | 1.662e-24 |
| 19 | 41503708 | rs7250991 | 0.53 | 0.05 | 1.662e-24 |
| 19 | 41504077 | rs16974799 | 0.53 | 0.05 | 1.662e-24 |
| 19 | 41504249 | rs62109049 | 0.53 | 0.05 | 1.662e-24 |
| 19 | 41524087 | rs707265 | -0.46 | 0.04 | 1.68e-24 |
| 19 | 41525374 | rs1552219 | -0.46 | 0.04 | 1.68e-24 |
| 19 | 41506665 | rs2200237 | 0.53 | 0.05 | 1.913e-24 |
| 19 | 41506964 | rs11881991 | 0.529 | 0.05 | 1.913e-24 |
| 19 | 41527085 | rs2086785 | -0.46 | 0.04 | 1.998e-24 |
| 19 | 41527265 | rs2200238 | -0.46 | 0.04 | 2.051e-24 |
| 19 | 41537538 | rs10404959 | -0.47 | 0.04 | 2.089e-24 |
| 19 | 41536622 | rs7257703 | -0.47 | 0.04 | 2.267e-24 |
| 19 | 41498348 | rs2099361 | -0.47 | 0.04 | 2.632e-24 |
| 19 | 41524009 | rs1038376 | 0.49 | 0.05 | 6.437e-24 |
| 19 | 41526144 | rs35131519 | 0.45 | 0.04 | 6.843e-24 |
| 19 | 41503404 | rs7250601 | 0.52 | 0.05 | 1.024e-23 |
| 19 | 41465130 | rs10417579 | -0.46 | 0.04 | 1.243e-23 |
| 19 | 41526239 | rs10423004 | 0.45 | 0.04 | 2.16e-23 |
| 19 | 41509438 | rs10403955 | 0.50 | 0.05 | 3.074e-23 |
| 19 | 41521939 | rs10853744 | 0.50 | 0.05 | 4.33e-23 |
| 19 | 41529677 | rs10421701 | 0.45 | 0.04 | 6.281e-23 |
| 19 | 41529781 | rs10420194 | 0.44 | 0.04 | 7.255e-23 |
| 19 | 41508442 | rs10500282 | 0.50 | 0.05 | 7.366e-23 |
| 19 | 41528206 | rs732738 | 0.44 | 0.04 | 8.281e-23 |
| 19 | 41531212 | rs3895941 | 0.43 | 0.04 | 1.302e-22 |
| 19 | 41503022 | rs4803416 | -0.44 | 0.04 | 3.099e-22 |
| 19 | 41468429 | rs988900 | -0.45 | 0.05 | 9.126e-22 |
| 19 | 41463593 | rs4803411 | -0.45 | 0.05 | 1.056e-21 |
| 19 | 41503460 | rs7250745 | 0.52 | 0.05 | 1.331e-21 |
| 19 | 41451810 | rs4560022 | -0.44 | 0.05 | 1.454e-21 |
| 19 | 41451893 | rs4560023 | -0.44 | 0.05 | 1.454e-21 |
| 19 | 41452293 | rs7251436 | -0.44 | 0.05 | 1.454e-21 |
| 19 | 41453582 | rs4239511 | -0.44 | 0.05 | 1.454e-21 |
| 19 | 41455816 | rs12972933 | -0.44 | 0.05 | 1.454e-21 |
| 19 | 41458785 | rs4322765 | -0.44 | 0.05 | 1.454e-21 |
| 19 | 41459241 | rs3889806 | -0.44 | 0.05 | 1.454e-21 |
| 19 | 41503447 | rs7251624 | 0.52 | 0.05 | 1.579e-21 |
| 19 | 41477303 | chr19:41477303:I | -0.44 | 0.05 | 2.359e-21 |
| 19 | 41531656 | rs7249735 | 0.46 | 0.05 | 5.207e-21 |
| 19 | 41476112 | rs150802555 | 0.54 | 0.06 | 6.147e-21 |
| 19 | 41530646 | rs10401226 | 0.46 | 0.05 | 8.219e-21 |
| 19 | 41531311 | rs11083596 | 0.46 | 0.05 | 8.219e-21 |
| 19 | 41531522 | rs11670865 | 0.46 | 0.05 | 8.219e-21 |
| 19 | 41531705 | rs3745275 | 0.45 | 0.05 | 1.117e-20 |
| 19 | 41451576 | rs4062238 | -0.43 | 0.05 | 5.082e-20 |
| 19 | 41450934 | rs55811526 | -0.44 | 0.05 | 5.403e-20 |
| 19 | 41520351 | rs7255374 | 0.44 | 0.05 | 1.424e-19 |
| 19 | 41529020 | rs7255904 | 0.39 | 0.04 | 7.708e-19 |
| 19 | 41448604 | rs12611133 | -0.42 | 0.05 | 1.715e-18 |
| 19 | 41448652 | rs55998109 | -0.42 | 0.05 | 1.715e-18 |
| 19 | 41449455 | rs11083593 | -0.41 | 0.05 | 3.466e-18 |
| 19 | 41465979 | rs11673114 | -0.40 | 0.05 | 8.845e-18 |
| 19 | 41474512 | rs10419680 | 0.42 | 0.05 | 1.517e-16 |
| 19 | 41531368 | rs11666982 | 0.37 | 0.04 | 1.553e-16 |
| 19 | 41465826 | chr19:41465826 | 0.42 | 0.05 | 3.143e-16 |
| 19 | 41467745 | rs10406402 | 0.41 | 0.05 | 4.201e-16 |
| 19 | 41469064 | rs1017384 | 0.41 | 0.05 | 4.577e-16 |
| 19 | 41465820 | chr19:41465820 | 0.41 | 0.05 | 5.028e-16 |
| 19 | 41464002 | rs10410037 | 0.41 | 0.05 | 5.48e-16 |
| 19 | 41472778 | rs7247273 | 0.41 | 0.05 | 6.484e-15 |
| 19 | 41458011 | chr19:41458011 | 0.40 | 0.05 | 1.154e-14 |
| 19 | 41456176 | rs1132886 | 0.39 | 0.05 | 1.644e-14 |
| 19 | 41453793 | rs7259806 | 0.39 | 0.05 | 1.67e-14 |
| 19 | 41458434 | rs71358950 | 0.39 | 0.05 | 2.057e-14 |
| 19 | 41448205 | rs8110485 | -0.35 | 0.05 | 8.766e-14 |
| 19 | 41460621 | rs8109818 | 0.37 | 0.05 | 2.071e-13 |
| 19 | 41454683 | rs7245500 | 0.36 | 0.05 | 2.827e-12 |
| 19 | 41536237 | rs10421834 | 0.44 | 0.06 | 5.336e-12 |
| 19 | 41532126 | rs7255146 | 0.42 | 0.06 | 2.138e-11 |
| 19 | 41532141 | chr19:41532141 | 0.42 | 0.06 | 2.138e-11 |
| 19 | 41532279 | rs11879790 | 0.42 | 0.06 | 2.138e-11 |
| 19 | 41532654 | rs10419321 | 0.42 | 0.06 | 2.138e-11 |
| 19 | 41532738 | rs10420699 | 0.42 | 0.06 | 2.138e-11 |
| 19 | 41532885 | rs10424716 | 0.42 | 0.06 | 2.138e-11 |
| 19 | 41533990 | rs8103372 | 0.42 | 0.06 | 2.138e-11 |
| 19 | 41534069 | rs8102557 | 0.42 | 0.06 | 2.138e-11 |
| 19 | 41534071 | rs8102771 | 0.42 | 0.06 | 2.138e-11 |
| 19 | 41534289 | rs8103097 | 0.42 | 0.06 | 2.138e-11 |
| 19 | 41534941 | rs28671673 | 0.42 | 0.06 | 2.138e-11 |
| 19 | 41534976 | rs10413524 | 0.42 | 0.06 | 2.138e-11 |
| 19 | 41535095 | rs10415473 | 0.42 | 0.06 | 2.138e-11 |
| 19 | 41535290 | rs11671743 | 0.42 | 0.06 | 2.138e-11 |
| 19 | 41535954 | rs16974878 | 0.42 | 0.06 | 2.138e-11 |
| 19 | 41536117 | rs10421597 | 0.42 | 0.06 | 2.138e-11 |
| 19 | 41536147 | rs10422829 | 0.42 | 0.06 | 2.138e-11 |
| 19 | 41536416 | rs10422282 | 0.42 | 0.06 | 2.138e-11 |
| 19 | 41536503 | rs10422151 | 0.42 | 0.06 | 2.228e-11 |
| 19 | 41536547 | rs10423743 | 0.42 | 0.06 | 2.228e-11 |
| 19 | 41536642 | rs10422729 | 0.42 | 0.06 | 2.228e-11 |
| 19 | 41536961 | rs10402037 | 0.42 | 0.06 | 2.228e-11 |
| 19 | 41537045 | rs10402611 | 0.42 | 0.06 | 2.228e-11 |
| 19 | 41537050 | rs10402070 | 0.42 | 0.06 | 2.228e-11 |
| 19 | 41537326 | rs10403268 | 0.42 | 0.06 | 2.228e-11 |
| 19 | 41537448 | rs10403140 | 0.42 | 0.06 | 2.228e-11 |
| 19 | 41537510 | rs10403330 | 0.42 | 0.06 | 2.228e-11 |
| 19 | 41537850 | rs10410867 | 0.42 | 0.06 | 2.228e-11 |
| 19 | 41537868 | rs10409701 | 0.42 | 0.064 | 2.228e-11 |
| 19 | 41537992 | rs10409738 | 0.42 | 0.06 | 2.228e-11 |
| 19 | 41538059 | rs10409585 | 0.42 | 0.06 | 2.228e-11 |
| 19 | 41538108 | rs10410347 | 0.42 | 0.06 | 2.228e-11 |
| 19 | 41538738 | rs1080235 | 0.42 | 0.06 | 2.228e-11 |
| 19 | 41538741 | rs1080234 | 0.42 | 0.06 | 2.228e-11 |
| 19 | 41539025 | rs1807967 | 0.42 | 0.06 | 2.228e-11 |
| 19 | 41539132 | rs10417678 | 0.42 | 0.06 | 2.228e-11 |
| 19 | 41539257 | rs10419098 | 0.42 | 0.06 | 2.228e-11 |
| 19 | 41546555 | rs55849132 | -0.38 | 0.06 | 2.426e-11 |
| 19 | 41548090 | rs11672352 | -0.38 | 0.06 | 3.698e-11 |
| 19 | 41535348 | rs11667928 | 0.43 | 0.06 | 4.127e-11 |
| 19 | 41552066 | rs11083601 | -0.38 | 0.06 | 5.403e-11 |
| 19 | 41554399 | rs56365287 | -0.37 | 0.06 | 5.799e-11 |
| 19 | 41494151 | rs79436912 | 0.52 | 0.08 | 6.127e-11 |
| 19 | 41546395 | rs58657125 | -0.37 | 0.06 | 7.348e-11 |
| 19 | 41545022 | rs7257660 | -0.37 | 0.06 | 8.841e-11 |
| 19 | 41544948 | rs7257645 | -0.37 | 0.06 | 1.069e-10 |
| 19 | 41545179 | rs11673238 | -0.37 | 0.06 | 1.072e-10 |
| 19 | 41539618 | rs10418426 | 0.41 | 0.06 | 1.142e-10 |
| 19 | 41555515 | rs8102336 | -0.36 | 0.06 | 1.485e-10 |
| 19 | 41556326 | rs62117378 | -0.36 | 0.06 | 1.648e-10 |
| 19 | 41560935 | rs55870512 | -0.35 | 0.05 | 1.666e-10 |
| 19 | 41533256 | rs11673685 | 0.41 | 0.06 | 1.769e-10 |
| 19 | 41558878 | rs56156262 | -0.36 | 0.06 | 1.9e-10 |
| 19 | 41560304 | rs7254075 | -0.36 | 0.06 | 2.249e-10 |
| 19 | 41539723 | rs10418657 | 0.40 | 0.06 | 3.132e-10 |
| 19 | 41550474 | rs58689390 | -0.32 | 0.05 | 5.517e-10 |
| 19 | 41558800 | rs11083604 | -0.32 | 0.05 | 6.117e-10 |
| 19 | 41559583 | rs28502605 | -0.32 | 0.05 | 6.568e-10 |
| 19 | 41539731 | rs10418473 | 0.40 | 0.06 | 6.93e-10 |
| 19 | 41555207 | rs8102824 | -0.33 | 0.05 | 6.941e-10 |
| 19 | 41557809 | chr19:41557809:D | -0.32 | 0.05 | 7.433e-10 |
| 19 | 41533009 | rs10426482 | 0.40 | 0.07 | 8.128e-10 |
| 19 | 41533028 | rs10424952 | 0.40 | 0.07 | 8.128e-10 |
| 19 | 41552040 | rs10853746 | -0.32 | 0.05 | 9.246e-10 |
| 19 | 41556935 | rs11083602 | -0.33 | 0.05 | 1.229e-09 |
| 19 | 41533049 | rs8192785 | 0.40 | 0.07 | 1.264e-09 |
| 19 | 41533087 | rs10426686 | 0.41 | 0.07 | 1.515e-09 |
| 19 | 41540285 | rs116340956 | 0.40 | 0.06 | 1.532e-09 |
| 19 | 41533112 | rs10425152 | 0.41 | 0.07 | 1.736e-09 |
| 19 | 41540351 | rs141106993 | 0.39 | 0.06 | 2.037e-09 |
| 19 | 41579158 | rs7259780 | -0.32 | 0.05 | 2.178e-09 |
| 19 | 41559582 | chr19:41559582:D | -0.30 | 0.05 | 3.193e-09 |
| 19 | 41539559 | rs10418063 | 0.40 | 0.07 | 3.408e-09 |
| 19 | 41572375 | rs11673698 | -0.31 | 0.05 | 3.883e-09 |
| 19 | 41539990 | rs10425932 | 0.40 | 0.07 | 3.927e-09 |
| 19 | 41531859 | rs7254767 | 0.37 | 0.06 | 4.169e-09 |
| 19 | 41564417 | rs12978001 | -0.31 | 0.05 | 5.629e-09 |
| 19 | 41566972 | rs11665820 | -0.31 | 0.05 | 5.893e-09 |
| 19 | 41568707 | rs8105353 | -0.31 | 0.05 | 5.893e-09 |
| 19 | 41569618 | rs7256289 | -0.31 | 0.05 | 5.893e-09 |
| 19 | 41571904 | rs7245443 | -0.31 | 0.05 | 5.893e-09 |
| 19 | 41573054 | rs8103299 | -0.31 | 0.05 | 5.996e-09 |
| 19 | 41573505 | rs11083606 | -0.31 | 0.05 | 5.996e-09 |
| 19 | 41540115 | rs10426184 | 0.40 | 0.07 | 6.52e-09 |
| 19 | 41587704 | rs7257232 | -0.31 | 0.05 | 6.774e-09 |
| 19 | 41589818 | rs7254343 | -0.31 | 0.05 | 6.774e-09 |
| 19 | 41540982 | rs145340105 | 0.39 | 0.07 | 8.35e-09 |
| 19 | 41533212 | rs10425384 | 0.38 | 0.07 | 8.362e-09 |
| 19 | 41540885 | rs146666608 | 0.39 | 0.07 | 8.367e-09 |
| 19 | 41532005 | rs434606 | 0.31 | 0.05 | 8.708e-09 |
| 19 | 41532133 | rs62120870 | 0.31 | 0.05 | 8.708e-09 |
| 19 | 41534806 | rs17726861 | 0.31 | 0.05 | 8.708e-09 |
| 19 | 41534901 | rs62120871 | 0.31 | 0.05 | 8.708e-09 |
| 19 | 41535701 | rs17799942 | 0.31 | 0.05 | 8.708e-09 |
| 19 | 41537698 | rs17726963 | 0.31 | 0.05 | 8.708e-09 |
| 19 | 41537962 | rs4803421 | 0.31 | 0.05 | 8.708e-09 |
| 19 | 41538033 | rs4802105 | 0.31 | 0.05 | 8.708e-09 |
| 19 | 41539479 | rs58511716 | 0.32 | 0.05 | 1.017e-08 |
| 19 | 41561980 | chr19:41561980 | -0.29 | 0.05 | 1.098e-08 |
| 19 | 41539283 | rs58788920 | 0.31 | 0.05 | 1.115e-08 |
| 19 | 41566543 | rs11083605 | -0.30 | 0.05 | 1.341e-08 |
| 19 | 41557290 | rs11083603 | -0.33 | 0.06 | 1.573e-08 |
| 19 | 41562218 | rs7255149 | -0.29 | 0.05 | 1.605e-08 |
| 19 | 41561676 | rs34533291 | -0.29 | 0.05 | 2.141e-08 |
| 19 | 41567316 | rs8100612 | -0.29 | 0.05 | 3.143e-08 |
| 19 | 41588024 | rs35206367 | -0.29 | 0.05 | 3.541e-08 |

**Supplementary** **table 2.** Results from a genome-wide methylation analysis for serum levels of *p,p’-*DDE. The Beta values are based on ln-transformed values of *p,p’-*DDE levels being used in the models. Only methylation sites with p-value < 10^-5^ are shown. e=*10^-^

| Methylation site | Beta | SE | p-value | Chromosome | Position | Gene |
| --- | --- | --- | --- | --- | --- | --- |
| cg27089200 | 0.12 | 0.021 | 6.2e-09 | 19 | 41531976 | CYP2B6 |
| cg07566625 | -0.088 | 0.018 | 3.2e-06 | 1 | 37980778 | MEAF6 |
| cg02681858 | -0.058 | 0.012 | 3.5e-06 | 2 | 39965301 | THUMPD2 |
| cg00318519 | -0.054 | 0.011 | 3.7e-06 | 2 | 37035707 | VIT |
| cg11991859 | -0.074 | 0.0164 | 5.9e-06 | 14 | 33408527 | NPAS3 |
| cg18753424 | 0.040 | 0.0090 | 6.4e-06 | 3 | 43026757 | FAM198A |
| cg17681447 | -0.043 | 0.0096 | 7.8e-06 | 7 | 6204302 | CYTH3 |
